# Systematic review with meta-analysis: cytokines in fibromyalgia syndrome

**DOI:** 10.1186/1471-2474-12-245

**Published:** 2011-10-28

**Authors:** Nurcan Üçeyler, Winfried Häuser, Claudia Sommer

**Affiliations:** 1 Department of Neurology, University of Würzburg, Josef-Schneider-Str. 11, 97080 Würzburg, Germany; 2 Center of Pain Therapy, Klinikum Saarbrücken gGmbH, Winterberg 1, 66119 Saarbrücken, Germany; 3 Department of Psychosomatic Medicine and Psychotherapie, Technische Universität München, Ismaningerstraße 22, 81675 München, Germany

## Abstract

**Background:**

To perform a systematic review and meta-analysis on cytokine levels in patients with fibromyalgia syndrome (FMS).

**Methods:**

Through December 2010 we systematically reviewed the databases PubMed, MEDLINE, and PsycINFO and screened the reference lists of 22 review articles for suitable original articles. Original articles investigating cytokines in patients with FMS were included. Data were extracted by two independent authors. Differences of the cytokine levels of FMS patients and controls were summarized by standardized mean differences (SMD) using a random effects model. Study quality was assessed applying methodological scores: modified Center of Evidence Based Medicine, Newcastle-Ottawa-Scale, and Würzburg Methodological Quality Score.

**Results:**

Twenty-five articles were included investigating 1255 FMS patients and 800 healthy controls. Data of 13/25 studies entered meta-analysis. The overall methodological quality of studies was low. The results of the majority of studies were not comparable because methods, investigated material, and investigated target cytokines differed. Systematic review of the selected 25 articles revealed that FMS patients had higher serum levels of interleukin (IL)-1 receptor antagonist, IL-6, and IL-8, and higher plasma levels of IL-8. Meta-analysis of eligible studies showed that FMS patients had higher plasma IL-6 levels compared to controls (SMD = -0.34 [-0.64, -0.03] 95% CI; p = 0.03). The majority of investigated cytokines were not different between patients and controls.

**Conclusions:**

The pathophysiological role of cytokines in FMS is still unclear. Studies of higher quality and with higher numbers of subjects are needed.

## Background

Fibromyalgia syndrome (FMS) is a complex of symptoms that, according to the criteria of the American College of Rheumatology (ACR), is associated with chronic widespread pain and at least eleven positive tender points out of 18 [1]. Additional symptoms like fatigue, pain associated sleep disturbance, depression, or gastrointestinal disorders are frequently reported [2]. The pathophysiology of FMS is incompletely understood and although the syndrome can be characterized by an identifiable group of signs and symptoms, there is no recognized etiologic agent and no consistent anatomical alteration that would qualify FMS as a disease (e.g. Stedman's online medical dictionary http://www.stedmans.com).

With an estimated prevalence of 0.5% to 5.8% in the general population of North America and Europe [3,4], FMS is a frequent condition and better pathophysiological knowledge is warranted to more effectively direct treatment. Although a large number of studies on the pathophysiology of FMS have been published, there is still no overall concept explaining all aspects of FMS. An imbalance of pro- and anti-inflammatory cytokines is assumed to play a role in the induction and maintenance of pain [5] and several laboratories have investigated cytokine levels in patients with FMS with diverse results. In non-systematic reviews the potential pathophysiological role of cytokines has been discussed with discrepant results [6,7]. A systematic review with a quantitative synthesis of the cytokine profiles of FMS patients compared to controls had not been performed until now. The aims of this systematic review therefore were to systematically and quantitatively review data on pro- and anti-inflammatory cytokines in FMS patients considering and assessing study quality.

## Methods

### Setting

The literature search was initiated as part of the development of an evidence-based interdisciplinary guideline for the diagnosis and therapy of FMS on behalf of the Association of the Scientific Medical Societies of Germany (AWMF) coordinated by the German Interdisciplinary Association of Pain (DIVS) [8]. The search was later expanded and extended (see below).

### Searches

A literature search for original research articles on the pathophysiology of FMS was performed through December 2010 and the following databases were screened: PubMed, MEDLINE, and PsycINFO. The keywords (all languages) "fibromyalgia", "fibromyalgia syndrome", "chronic widespread pain" were used in combination with "cytokine", with "cytokine" AND "pathophysiology", and also with "review". Additionally, the reference sections of 22 review articles that were found using this search strategy were screened for possibly suitable original articles.

### Inclusion and exclusion criteria

We only included original full text articles investigating the pathophysiological role of cytokines in FMS and excluded the following articles that appeared upon the search strategy detailed above: double hits, reviews, letters to the editor, papers investigating other disorders, articles in Danish, all articles that did not investigate cytokines. First, the titles and abstracts of all articles found were screened for suitability; the initially chosen articles were then screened again checking the entire article.

### Data extraction

Two authors independently screened the titles and abstracts of potentially eligible articles that were detected by the search strategy described above. The full text of the selected studies was examined. For data extraction two authors (NÜ, CS) independently used standard extraction forms. All authors cross-checked the extraction forms for correctness. Data were categorized for the material investigated. Blood measurements were performed in serum, plasma, and whole blood; accordingly these data were first assessed separately and then all study results on "blood" were compared.

### Quality assessment

For evaluating the quality of the selected studies we applied two validated scales: the Level of Evidence of the Oxford Center for Evidence-based Medicine (CEBM, http://www.cebm.net/index.aspx?o=1025) and the Newcastle-Ottawa Quality Assessment Scale (NOS) http://www.ohri.ca/programs/clinical_epidemiology/oxford.asp.

We modified the CEBM to make it applicable for scientific papers investigating pathophysiological aspects with bench research (Table 1). Additionally, we aimed at evaluating the quality of the laboratory methods used. To our knowledge there is no standardized rating scale to assess the quality of laboratory methods in scientific papers. Therefore we created the Würzburg Methodological Quality Score (W-MeQS, Table 2). W-MeQS consists of twelve items that are relevant for high quality of a laboratory method. For each item fulfilled one point is given; the score is the sum of all points achieved divided by the number of items that were applicable. If the target of interest was measured by just one method this score marks the end score. If more than one method was used, a subscore is calculated for each method as described and the end-score is calculated as mean of the subscores. The scores were evaluated as follows: ≤ 0.4: low quality; > 0.4 medium quality; ≥ 0.8 high quality.

**Table 1:** Levels of CEBM modified for scientific papers investigating disease pathophysiology with bench research.

Level	Differential diagnosis/symptom prevalence study	Blinded	Controls	Remark/example
1a	Systematic review of prospective cohort studies	NR	NR	No systematic reviews available

1b	Prospective cohort study with good follow-up	NR	Implicit	An item is assessed (e.g. breast implant) and the occurrence of FMS is followed prospectively

1c	All or none case series	Test and assessment of results	Obligatory	All patients with low IL-4 have FMS, none has high IL-4

1d	1c	Test or assessment of results	Obligatory	1c

2a	Systematic review (with homogeneity) of	NR	NR	No systematic reviews available

	2b and better studies			

2b	Retrospective cohort study, or poor follow-up	NR	Implicit	FMS patients are investigated now, if they have had breast implants in the past; large size studies

2c	Ecological studies	Test and assessment of results	Implicit	FMS patients are investigated simultaneously, if they have FMS and if they have low IL-4 levels.

2d	2c	Test or assessment of results	Implicit	2c

3a	Systematic review (with homogeneity) of 3b and better studies			No systematic reviews available

3b	Non-consecutive cohort study, or very limited population	Test and assessment of results	Obligatory	FMS patients and controls (limited population) are now investigated if they have low IL-4

				levels

3c	3b	Test or assessment of results	Obligatory	3b

3d	1c, 2c, 3b	Not blinded	Obligatory	

4	Case series or superseded reference standards		All studies without control group	Case series with historical controls; case reports

5	Expert opinion without explicit critical appraisal, or based on physiology, bench research or "first principles"	NR		

**Abbreviations: **CEMB: Oxford Center for Evidence-based Medicine FMS: fibromyalgia syndrome; NR: not relevant

**Table 2:** Würzburg Methodological Quality Score (W-MeQS) for the assessment of the quality of laboratory methods.

	Item
1	Standardized method OR citation OR new method with adequate description including 2-5?

2	Adequate description of the method itself?

3	Sensitivity limits of the method declared?

4	Adequate internal controls measured?

5	Adequate negative controls measured?

6	Adequate number of samples investigated?

7	Adequate comparison with the control group?

8	All reagent information given?

9	Time points of tissue/blood collection standardized and given?

10	Measurements or data assessment performed in a blinded manner?

11	Adequate data assessment and illustration?

12	Target of interest investigated with > 1 method?

### Data synthesis and analysis

Meta-analysis was performed using RevMan analysis software (RevMan 5.1.2) of the Cochrane Collaboration [9]. Standardized mean differences (SMD) were calculated by means and standard deviations for each cytokine.

## Results

### Study selection

**Figure 1:**
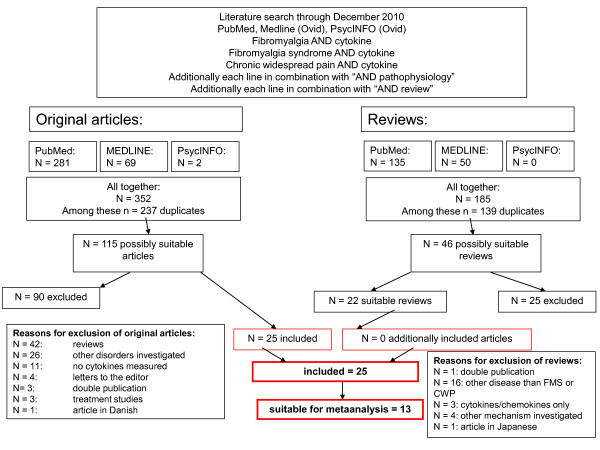
**Algorithm of study selection**. The Figure displays the details of the study search and selection process.

For the number of found, selected, excluded and finally included articles see the algorithm shown in Figure 1.

The initial literature search retrieved the following numbers of articles: PubMed: 281; MEDLINE: 69; PsycINFO: 2. After exclusion of double hits 115 articles were chosen and the titles and abstracts were screened for suitability. The following 90 articles were excluded: reviews (n = 42), papers investigating other disorders (n = 26), papers not investigating cytokines (n = 11), letters to the editor (n = 4), double publications (n = 3), treatment studies (n = 3), and one article in Danish. The screening of the reference sections of 22 review articles did not provide further original articles. Finally, 25 articles were selected for our ***systematic review ***[10-34]. A total of 1255 patients with FMS, 180 patients in disease control groups, and 800 healthy controls were investigated.

Of these 25 articles, two articles were not suitable for meta-analysis because no healthy control group was investigated [11,26]; seven articles could not be included, because mean and standard deviations were not given in the article or could not be calculated (e.g. from the standard error of the mean) and were not provided upon request [10,13,15,20,21,25,27]. Finally, 16 articles were principally suitable for ***meta-analysis ***[12,14,16-19,22-24,28-34]. These articles were then screened for suitability to be categorized in groups of articles having investigated the same cytokine with the same method in the same material; three of the 16 articles could not be categorized in one of the subgroups (see below) and therefore could not enter meta-analysis [12,18,33]. Thus, 13 articles were finally included in the meta-analysis. Table 3 summarizes key information of the 25 selected articles and gives the quality scores of the studies.

**Table 3:** Characteristics of all studies included in the systematic review investigating cytokines in FMS patients.

**Author, yr**. Diagnosis criteria	N patients/controls	a) Material b) Methods c) Investigated targets	Results	Modified CEBM level	NOS	W-MeQS
Hader, 1991 Smythe	12/10	a) CD4+ T-lymphocytes from PBMC b) T-cell culture; stimulation experiments with mitogens and measurement of IL-2 secretion c) IL-2	FMS: higher concentration of mitogen was necessary to achieve optimal IL-2 secretion; peak time of IL-2 secretion was delayed. Addition of calcium did not correct the reduction in IL-2 secretion in patients with FMS; addition of phorbole myristate acetate led to normal IL-2 secretion.	3d	2	0.4

Barth, 1999 Wolfe, 1985	12 FMS/6 rheumatoid arthritis or osteoarthritis controls/6 controls	a) supernatant of PBMC b) self established double sandwich ELISA; c) IL-4; IL-2; INFγ; GM-CSF; IL-5, IL-10	In vitro stimulation of PBMC with different L-tryptophan preparations: 6/12 FMS patients, 2/12 controls: IL-5 and IL-10 production	3d	4	0.1

Maes, 1999 ACR	21/33	a) serum B) ELISA c) IL-6, sIL-6 R, sIL-1R, IL-1RA	In FMS compared to controls: IL-6↔ sIL-6R ⇑ sIL-1R ⇑ IL-1RA ⇑	3d	2	0.3

Pay. 2000 ACR	25 FMS/25 chronic musculoskeletal complaints/25 controls	a) serum b) ELISA c) IL-1β, TNF, IL-6	No difference for pro-inflammatory cytokines in FMS and controls.	3d	3	0.4

Wallace, 2001 ACR	56/56 Serum, PBMC	a) serum, PBMC, plasma b) ELISA c) IL-1β, IL-2, IL-6, IL-8, IL-10, sIL-2R, IL-1RA, IFNγ, TNF	In FMS compared to controls: IL-1β, IL-2, IL-6, IL-8, IL-10, sIL-2R, IFNγ, TNF: ↔ in sera +PBMC IL-1RA: ⇑ in serum IL-8: ⇑ in plasma IL-1 RA, IL-6: ⇑ in PBMC IL-6: ⇑ in PBMC of patients with disease duration > 2 years.	3d	3	0.5

Gür, 2002 ACR	81/32	a) serum b) ELISA c) IL-1, IL-2R, IL-6, IL-8	In FMS compared to controls: IL-1 ↔ IL-2 R ⇑ IL-6 ↔ IL-8 ⇑	3d	2	0.4

Schwarz, 2002 ACR	17/17	a) serum b) ELISA c) IL-6	IL-6 ⇑ during tryptophan depletion in FMS	3d	4	0.3

Amel Kashipaz, 2003 ACR	22 FMS/CFS/19	a) PBMC b) intracellular cytokine stain; flow cytometry c) IL-1α, IL-6, IL-10, TNF	In FMS compared to controls: IL-1α ↔ IL-6 ↔ IL-10 ↔ TNF ↔	3d	2	0.7

Salemi, 2003 ACR	53/10	a) skin biopsy b) RT-PCR, IHC c) IL-1β, IL-6, TNF	Detectable cytokines in FMS: IL-1β (19/50) IL-6 (14/51) TNF (17/53) None of the cytokines could be detected in control skin.	3d	2	0.7

Ardic, 2006 ACR	21/10	a) serum b) ELISA c) IL-1 (after balneo therapy)	After balneo therapy: IL-1↓ in FMS	3d	3	0.2

Üçeyler, 2006 ACR	26/40	a) serum; whole blood b) qRT-PCR; ELISA c) IL-2, IL-4, IL-8, IL-10, TNF, TGF-β1	In FMS compared to controls: IL-2 ↔ IL-4 ⇓ IL-8 ↔ IL-10 ⇓ TGF-β1 ↔ TNF ↔	3d	4	0.8

Bazzichi, 2007 ACR	285/40 (16 rheumatoid arthritis cases, two Sjögren's syndrome cases, 16 systemic lupus erythematosus cases, four systemic sclerosis cases, two undifferentiated connective- tissue disease cases)/100	a) serum, plasma b) ELISA c) IL-1, IL-6, IL-8, IL-10, TNFα	No intergroup difference for cytokines.	3d	3	0.2

Bazzichi, 2007 ACR	80/45	a) plasma b) ELISA c) IL-1, IL-6, IL-8, IL-10, TNF	IL-10, IL-8, TNF: FMS > controls	3c	3	0.9

Macedo, 2007 ACR	18/22	a) PBMC b) automated biochip array; before and after 1.5 mg of dexamethasone per os c) IL-1α, IL-1β, IL-2, IL-4, IL-6, IL-8, IL-10, IFNγ, TNF	After dexamethasone: reduction of cytokines FMS > controls.	3d	2	0.4

Kaufmann, 2007 ACR	22/15 CRPS/37	a) T-cells b) FACS analysis c) IL-2, IFNγ, IL-4, IL-10	No difference in percentage of cytokine producing cells between FMS and controls.	3d	2	0.6

Togo, 2008 ACR	7/9	a) plasma b) Beadlyte multi-cytokine assay c) IL-10, IL-6, IL-8, IL-1, TNF	No difference between groups. "FM patients showed a shift to increased IL-10 in the nighttime compared to controls."	3d	2	0.8

Wang, 2008 ACR	20/80	a) serum b) Bio-Plex cytokine assay c) IL-6, IL-8, IL-10, IL-4, TNF	At baseline: IL-8 in FMS > controls; no difference for other cytokines.	3d	4	0.4

Zhang, 2008 ACR	92/69 family members/62 anonymous blood samples from blood bank	a) plasma b) Cytokine Twenty-Five-Plex Antibody Bead Kit c) MCP-1, Eotaxin, IP-10, IL-13, IL-5, IL-10, IL-1b, IL-2, IL-4, IL-6, IL-7, IL-8, IL-12, IL-15, IL-17, TNF, IFNa, IFNg, GM-CSF, MIG, MIP-1a, MIP-1b, IL-1ra, IL-2r	Eotaxin and MIP: FMS > controls	3d	3	0.5

Feng, 2009 ACR	100 FMS patients and family members/35 unaffected parents	a) plasma b) Cytokine Twenty-Five-Plex Antibody Bead Kit c) Eotaxin, MIP.1a, MCP-1, IP10, IL-12, IL-1β	Rare missense variants of the MEFV gene are associated with risk of FMS and are present in a subset of 15% of FMS patients. This subset had, on average, high levels of plasma IL-1b compared to FMS patients without rare variants, unaffected family members with or without rare variants, and unrelated controls of unknown genotype.	3d	3	0.4

Blanco, 2010 ACR	63/49	a) skin b) immuno-histochemistry c) MCP-1, TNF	MCP-1: FMS < controls	3c	3	0.8

Blanco, 2010 ACR	79/59	a) plasma b) sandwich enzyme immunoassay kits c) IL-8, TNF, sTNF-RI, sTNF-RII, MCP-1	Patients with FMS have lower systemic levels of MCP-2 than controls.	3d	3	0.4

Hernandez, 2010 ACR	64/25	a) serum b) ELISA c) TNF, IL-1, IL-6	TNF: FMS < controls IL-1: not detectable in FMS IL-6: FMS > controls	3c	4	0.6

Iannucelli, 2010 ACR	51/25 tension type headache/15	a) serum b) multiplex bead-based sandwich immunoassay c) IL-1β, IL-1Rα, IL-4, IL-6, IL-8, IL-10, INFγ, TNF	FMS > controls: IL-1RA, IL-6, IL-10, TNF	3d	3	0.7

Ortega, 2010 ACR	9/9	a) PBMC b) ELISA c) IL-1β, TNF, IL-6, IL-10	For all cytokines investigated: higher values at baseline in FMS compared to controls; after aquatic exercise levels as in controls.	3d	3	0.3

Ross, 2010 ACR	24/none	a) serum b) bead-based immunofluorescence assay c) IL-1α, IL-1β, IL-1RA, IL-6, IL-8, IL-10, TNF	IL-6 and IL-8: FMS responders (i.e. GH response to exercise of ≥ 5 ng/mL) higher than FMS non-responders. For IL-1α vice versa.	4	1	0.2

Abbreviations:

ACR: American College of Rheumatology; CEMB: Center of Evidence Based Medicine; ELISA: enzyme linked immunosorbent assay; FMS: fibromyalgia syndrome; IL: interleukin; NOS: Newcastle Ottawa Scale; NR: not reported; PBMC: peripheral blood mononuclear cells; qRT-PCR: quantitative real-time PCR; R: receptor; RA: receptor antagonist; W-MeQS: Würzburg Methodological Quality Score; yrs: years

### Majority of studies with low quality

Only three of the 25 studies (12%) had a modified CEBM level of 3c [14,15,19]. Twenty-one of the 25 (84%) studies had a modified CEBM level of 3d [10-13,16-18,20-25,27-34], and one case report of 4 (4%) [26]. The highest NOS score reached was 4 out of 8; this was achieved by 5/25 studies (25%) [12,28,30,32]. Eleven out of 25 studies (44%) reached a NOS score of 3 [11,13-17,20,24,25,31,33]; 8/25 studies (32%) had a NOS score of 2 [10,18,21-23,27,29,34]; one study had a NOS score of 1 (4%) [26].

In the W-MeQS only 4/25 studies (16%) were of high methodological quality (score ≥ 0.8) [14,15,29,30]; 7/25 studies (28%) were of medium quality (score > 0.4) [10,19-21,27,31,33], and 14/25 studies (56%) were of low quality (score ≤ 0.4) [11-13,16-18,22-26,28,32]. Only 2/25 studies (8%) had a high NOS and W-MeQS score [14,15].

These results show that the majority of the studies were of low methodological quality.

### High variability of materials and methods

In twelve studies serum [11,13,19,20,23,25,26,28,30-32,34], in seven studies peripheral blood mononuclear cells (PBMC) [10,12,18,21,22,24,31], in six studies plasma [13,14,16,17,29,33], in two studies skin biopsies [15,27], and in one study whole blood [30] was investigated. Cytokine levels were investigated with ELISA in twelve studies [11-14,19,23-25,28,30,31,34], with bead-based immunoassays in five studies [17,20,26,29,33], with FACS analysis in two studies [10,21], with quantitative real-time PCR in one study [30], with reverse transcription PCR in one study [27], with immunohistochemistry in one study [15].

There is little overlap in materials and methods used between different studies investigating cytokine levels in FMS patients.

### Systematic review

#### Serum (Table 4)

Serum samples of 643 FMS patients, 109 patients in disease control groups, and 301 healthy controls were investigated. Only one study reached a modified CEMB level of 3c [19]; ten studies had a modified CEMB level of 3d [11,13,20,23,25,28,30-32,34]; one study had a modified CEMB level of 4 [26]. Four studies had a NOS score of 4 [19,28,30,32], five studies had a NOS score of 3 [11,13,20,25,31], two studies had a NOS score of 2 [23,34], and one study of 1 [26]. Applying W-MeQS only one study was of high methodological quality [30]; three studies were of medium quality [19,20,31], and eight studies of low methodological quality [11,13,23,25,26,28,32,34].

**Table 4:** Characteristics of studies investigating serum levels of cytokines in FMS patients.

**Author, yr**. Diagnosis criteria	N patients/controls	a) Material b) Methods c) Investigated targets	Results	Modified CEBM level	NOS	W-MeQS
Maes, 1999 ACR	21/33	a) serum B) ELISA c) IL-6, sIL-6 R, sIL-1R, IL-1 RA	In FMS compared to controls: IL-6↔ sIL-6 R ⇑ sIL-1R ⇑ IL-1 RA ⇑	3d	2	0.3

Pay. 2000 ACR	25 FMS/25 chronic musculoskeletal complaints/25 controls	a) serum b) ELISA c) IL-1b, TNF, IL-6	No difference for pro-inflammatory cytokines in FMS and controls.	3d	3	0.4

Wallace, 2001 ACR	56/56	a) serum, PBMC, plasma b) ELISA c) IL-1β, IL-2, IL-6, IL-8, IL-10, sIL-2 R, IL-1 RA, IFNγ, TNF	In FMS compared to controls: IL-1β, IL-2, IL-6, IL-8, IL-10, sIL-2 R, IFNγ, TNF: ↔ in sera +PBMC IL-1 RA: ⇑ in serum IL-8: ⇑ in plasma IL-1 RA, IL-6: ⇑ in PBMC IL-6: ⇑ in PBMC of patients with disease duration > 2 years.	3d	3	0.5

Gür, 2002 ACR	81/32	a) serum b) ELISA c) IL-1, IL-2R, IL-6, IL-8	In FMS compared to controls: IL-1 ↔ IL-2 R ⇑ IL-6 ↔ IL-8 ⇑	3d	2	0.4

Schwarz, 2002 ACR	17/17	a) serum b) ELISA c) IL-6	IL-6 ⇑ during tryptophan depletion in FMS	3d	4	0.3

Ardic, 2006 ACR	21/10 (data not given)	a) serum b) ELISA c) IL-1 (after balneo therapy)	After balneo therapy: IL-1↓ in FMS	3d	3	0.2

Üçeyler, 2006 ACR	26/40	a) serum; whole blood b) qRT-PCR; ELISA c) IL-2, IL-4, IL-8, IL-10, TNF, TGF β1	In FMS compared to controls: IL-2 ↔ IL-4 ⇓ IL-8 ↔ IL-10 ⇓ TGF β1 ↔ TNF ↔	3d	4	0.8

Bazzichi, 2007 ACR	285/40 (16 rheumatoid arthritis cases, two Sjögren's syndrome cases, 16 systemic lupus erythematosus cases, four systemic sclerosis cases, two undifferentiated connective- tissue disease cases)/100	a) serum, plasma b) ELISA c) IL-1, IL-6, IL-8, IL-10, TNF	No intergroup difference for cytokines.	3d	3	0.2

Wang, 2008 ACR	20/80	a) serum b) Bio-Plex cytokine assay c) IL-6, IL-8, IL-10, IL-4, TNF	At baseline: IL-8 in FMS > controls; no difference for other cytokines.	3d	4	0.4

Hernandez, 2010 ACR	64/25	a) serum b) ELISA c) TNF, IL-1, IL-6	TNF: FMS < controls IL-1: not detectable in FMS IL-6: FMS > controls	3c	4	0.6

Iannucelli, 2010 ACR	51/25 tension type headache/15	a) serum b) multiplex bead-based sandwich immunoassay c) IL-1b, IL-1Ra, IL-4, IL-6, IL-8, IL-10, INFγ, TNF	FMS > controls: IL-1Ra, IL-6, IL-10, TNF	3d	3	0.7

Ross, 2010 ACR	24/none	a) serum b) bead-based immunofluorescence assay c) IL-1a, IL-1b, IL-1RA, IL-6, IL-8, IL-10, TNF	IL-6 and IL-8: FMS responders (i.e. GH response to exercise of ≥ 5 ng/mL) higher than FMS non-responders. For IL-1a vice versa.	4	1	0.2

FMS > controls

IL-1 RA: Iannuccelli 2010; Maes, 1999; Wallace 2001

IL-6: Iannucelli, 2010; Schwarz, 2002; Hernandez, 2010

IL-8: Gür, 2002; Wang, 2008

FMS = controls

IL-6: Maes, 1999; Pay, 2000; Wallace, 2001; Gür, 2002b; Bazzichi, 2007; Wang 2008

IL-1b/IL-1: Pay, 2000; Wallace, 2001; Gür, 2002b; Bazzichi, 2007; Iannucelli, 2010

TNF: Pay, 2000; Wallace, 2001; Bazzichi, 2007; Wang, 2008

IL-2: Wallace, 2001; Üçeyler, 2006

IL-10: Wallace, 2001; Bazzichi, 2007; Wang, 2008

IFN-g: Wallace, 2001; Iannucelli, 2010

IL-4: Wang, 2008; Iannucelli, 2010

Abbreviations:

ACR: American College of Rheumatology; CEMB: Center of Evidence Based Medicine; ELISA: enzyme linked immunosorbent assay; FMS: fibromyalgia syndrome; IL: interleukin; KYN: kynurenine; NOS: Newcastle Ottawa Scale; NR: not reported; PBMC: peripheral blood mononuclear cells; qRT-PCR: quantitative real-time PCR; R: receptor; RA: receptor antagonist; W-MeQS: Würzburg Methodological Quality Score; yr: year

The following results were reproduced (regardless of the method used and with a minimum of two independent studies): patients with ***FMS have higher serum levels ***of IL-1RA [20,23,31], IL-6 [19,20,28], and IL-8 [32,34]. ***No intergroup difference ***was found for serum IL-6 [13,23,25,31,32,34], IL-1β/IL-1 [13,20,25,31,34], TNF [13,25,30-32], IL-2 [30,31], IL-10 [13,31,32], IFN-γ [20,31], IL-4 [20,32].

Discrepant results are described for IL-6: IL-6 levels were found higher in FMS patients compared to controls in three studies [19,20,28]; 2/3 studies were of medium methodological quality [19,20], 2/3 studies had a NOS score of 4 [19,28], and 1/3 study had a modified CEBM of 3c [19]. In six studies no intergroup difference could be found for serum IL-6 levels [13,23,25,31,32,34]; 5/6 studies were of low methodological quality [13,23,25,32,34], only 1/6 studies reached a NOS score of 4 [32], and 6/6 studies had a modified CEBM level of 3d [13,23,25,31,32,34]. The better quality of studies showing elevated serum IL-6 levels in FMS patients compared to controls favors this finding, although in more studies (with less quality) no intergroup difference could be found.

The majority of studies were of low methodological quality and there was hardly an overlap in the methods used and the cytokines investigated. Only IL-1RA, IL-6, and IL-8 were higher expressed in sera of FMS patients, while for all other cytokines no intergroup difference could be found.

#### Plasma (Table 5)

Plasma cytokine levels were investigated in seven studies [13,14,16,17,29,31,33].

**Table 5:** Characteristics of studies investigating plasma levels of cytokines in FMS patients.

**Author, yr**. Diagnosis criteria	N patients/controls	a) Material b) Methods c) Investigated targets	Results	Modified CEBM level	NOS	W-MeQS
Wallace, 2001 ACR	56/56	a) serum, PBMC, plasma b) ELISA c) IL-1β, IL-2, IL-6, IL-8, IL-10, sIL-2R, IL-1RA, IFNγ, TNF	In FMS compared to controls: IL-1β, IL-2, IL-6, IL-8, IL-10, sIL-2R, IFNγ, TNF: ↔ in sera +PBMC IL-1RA: ⇑ in serum IL-8: ⇑ in plasma IL-1RA, IL-6: ⇑ in PBMC IL-6: ⇑ in PBMC of patients with disease duration > 2 years.	3d	3	0.5

Bazzichi, 2007 ACR	285/40 (16 rheumatoid arthritis cases, two Sjögren's syndrome cases, 16 systemic lupus erythematosus cases, four systemic sclerosis cases, two undifferentiated connective- tissue disease cases)/100	a) serum, plasma b) ELISA c) IL-1, IL-6, IL-8, IL-10, TNF	No intergroup difference for cytokines.	3d	3	0.2

Bazzichi, 2007 ACR	80/45	a) plasma b) ELISA c) IL-1, IL-6, IL-8, IL-10, TNF	IL-10, IL-8, TNF: FMS > controls	3c	3	0.9

Togo, 2008 ACR	7/9	a) plasma b) Beadlyte multi-cytokine assay c) IL-10, IL-6, IL-8, IL-1, TNF	No difference between groups.	3d	2	0.8

Zhang, 2008 ACR	92/69 family members/62 anonymous blood samples from blood bank	a) plasma b) Cytokine Twenty-Five-Plex Antibody Bead Kit c) MCP-1, Eotaxin, IP-10, IL-13, IL-5, IL-10, IL-1β, IL-2, IL-4, IL-6, IL-7, IL-8, IL-12, IL-15, IL-17, TNF, IFNα, IFNγ, GM-CSF, MIG, MIP-1α, MIP-1β, IL-1RA, IL-2R	Eotaxin and MIP: FMS > controls	3d	3	0.5

Feng, 2009 ACR	100 FMS patients and family members/35 unaffected parents	a) plasma b) Cytokine Twenty-Five-Plex Antibody Bead Kit c) Eotaxin, MIP-1α, MCP-1, IP10, IL-12, IL-1β	Rare missense variants of the MEFV gene are associated with risk of FMS and are present in a subset of 15% of FMS patients. This subset had, on average, high levels of plasma IL-1b compared to FMS patients without rare variants, unaffected family members with or without rare variants, and unrelated controls of unknown genotype.	3d	3	0.4

Blanco, 2010 ACR	79/59	a) plasma b) sandwich enzyme immunoassay kits c) IL-8, TNF, sTNF-RI, sTNF-RII, MCP-1	Patients with FMS have lower systemic levels of MCP-2 than controls.	3d	3	0.4

FMS > controls

IL-8: Bazzichi, 2007b; Wallace, 2001

FMS = controls

IL-1β/IL-1: Wallance, 2001; Bazzichi, 2007a and b; Zhang, 2008; Feng, 2009; Togo, 2009

IL-6: Wallance, 2001; Bazzichi, 2007a; Bazzichi, 2007a and b; Zhang, 2008; Togo, 2009

IL-8: Bazzichi, 2007a; Zhang, 2008; Togo, 2009; Blanco, 2010b

TNF: Wallance, 2001; Bazzichi, 2007a; Zhang, 2008; Togo, 2009; Blanco, 2010b

IL-10: Wallace, 2001; Bazzichi, 2007a; Zhang, 2008; Togo, 2009

MCP-1 and IP10: Zhang, 2001;Feng 2009

IL-2: Wallace, 2001; Zhang, 2008

IFNγ: Wallace, 2001; Zhang, 2008

Abbreviations:

ACR: American College of Rheumatology; CEMB: Center of Evidence Based Medicine; ELISA: enzyme linked immunosorbent assay; FMS: fibromyalgia syndrome; IL: interleukin; NOS: Newcastle Ottawa Scale; NR: not reported; PBMC: peripheral blood mononuclear cells; R: receptor; RA: receptor antagonist; W-MeQS: Würzburg Methodological Quality Score; yr: year

Plasma samples of 699 FMS patients, 109 patients in disease control groups, and 357 healthy controls were investigated. Only one study reached a modified CEMB level of 3c [14]; six studies had a modified CEMB level of 3d [13,16,17,29,31,33]. Six studies had a NOS score of 3 [13,14,16,17,31,33], one study of 2 [29]. Applying W-MeQS only two studies were of high methodological quality [14,29], two studies were of medium [31,33], and two studies of low methodological quality [16,17].

The following results were reproduced (regardless of the method used and with a minimum of two independent studies): patients with ***FMS have higher plasma levels ***of IL-8 [14,31]. ***No intergroup difference ***was found for plasma IL-1β/IL-1 [13,14,17,29,31,33], IL-6 [13,14,29,31,33], IL-8 [13,16,29,33], TNF [13,16,29,31,33], and IL-10 [13,29,31,33], MCP-1 and IP10 [17,33], IL-2 and IFN-γ [31,33].

Discrepant results are described for IL-8: IL-8 levels were found higher in FMS patients compared to controls in two studies [14,31]; 1/2 studies was of medium [31] and 1/2 studies was of high methodological quality [14], both studies had a NOS score of 3, and one study reached a modified CEBM level of 3c [14]. In four studies no intergroup difference could be found for plasma IL-8 levels [13,16,29,33]; 2/4 studies were of low methodological quality [13,16] and 3/4 studies had a NOS score of 3 [13,16,33]; the only study with high methodological quality had a NOS score of only 2 [29]; all four studies had a modified CEBM level of 3d. The better quality of studies showing elevated plasma IL-8 levels in FMS patients compared to controls favors this finding, although in more studies (with less quality) no intergroup difference could be found.

The majority of studies were of low methodological quality with only little overlap in methodology and cytokines investigated. A difference between FMS patients and controls was found only for IL-8 plasma levels. When taking studies investigating serum and plasma together we found no intergroup difference.

#### Whole blood (Table 6)

Cytokine expression in whole blood of patients with FMS was investigated in one study including 26 FMS patients and 40 healthy controls [30]. The study had a modified CEMB level of 3d, a NOS of 4 and was of high methodological quality and found reduced levels of the anti-inflammatory cytokines IL-4 and IL-10 gene expression in FMS patients compared to controls. These results were not reproduced.

**Table 6:** Characteristics of studies investigating whole blood levels of cytokines in FMS patients.

**Author, yr**. Diagnosis criteria	N patients/controls	a) Material b) Methods c) Investigated targets	Results	Modified CEBM level	NOS	W-MeQS
Üçeyler, 2006 ACR	26/40	a) serum; whole blood b) qRT-PCR; ELISA c) IL-2, IL-4, IL-8, IL-10, TNF, TGF- β1	In FMS compared to controls: IL-2 ↔ IL-4 ⇓ IL-8 ↔ IL-10 ⇓ TGF-β1 ↔ TNF ↔	3d	4	0.8

Abbreviations:

ACR: American College of Rheumatology; CEMB: Center of Evidence Based Medicine; ELISA: enzyme linked immunosorbent assay; FMS: fibromyalgia syndrome; IL: interleukin; NOS: Newcastle Ottawa Scale; qRT-PCR: quantitative real-time PCR; W-MeQS: Würzburg Methodological Quality Score; yr: year

#### Peripheral blood mononuclear cells (Table 7)

Cytokine expression in peripheral blood mononuclear cells (PBMC) was investigated in seven studies [10,12,18,21,22,24,31].

**Table 7:** Characteristics of studies investigating levels of cytokines in PBMC of FMS patients.

**Author, yr**. Diagnosis criteria	N patients/controls	a) Material b) Methods c) Investigated targets	Results	Modified CEBM level	NOS	W-MeQS
Hader, 1991 Smythe	12/10	a) CD4+ T-lymphocytes from PBMC b) T-cell culture; stimulation experiments with mitogens c) IL-2	FMS: higher concentration of mitogen was necessary to achieve optimal IL-2 secretion; peak time of IL-2 secretion was delayed. Addition of calcium did not correct the reduction in IL-2 secretion in patients with FMS; addition of phorbole myristate acetate led to normal IL-2 secretion.	3d	2	0.4

Barth, 1999 cite Wolfe, 1985	12 FMS/6 rheumatoid arthritis or osteoarthritis controls/6	a) supernatant of PBMC b) in vitro stimulation of PBMC with different L-tryptophan, contaminated L-tryptophan, peak E; self established double sandwich ELISA c) IL-4, IL-2, INF-γ, GM-CSF, IL-5, IL-10	In vitro stimulation of PBMC with different L-tryptophan preparations: 6/12 FMS patients, 2/12 controls: IL-5 and IL-10 production	3d	4	0.1

Wallace, 2001 ACR	56/56	a) serum, PBMC b) ELISA c) IL-1β, IL-2, IL-6, IL-8, IL-10, sIL-2 R, IL-1 RA, IFNγ, TNF	In FMS compared to controls: IL-1β, IL-2, IL-10, sIL-2 R, IFNγ, TNF: ↔ in sera + PBMC IL-1 RA, IL-8: ⇑ in sera IL-1 RA, IL-6: ⇑ in PBMC IL-6: ⇑ in PBMC of patients with disease duration > 2 years.	3d	3	0.5

Amel Kashipaz, 2003 ACR	22 FMS/CFS/19	a) PBMC b) intracellular cytokine stain; flow cytometry c) IL-1α, IL-6, IL-10, TNF	In FMS compared to controls: IL-1α ↔ IL-6 ↔ IL-10 ↔ TNF ↔	3d	2	0.7

Kaufmann, 2007 ACR	22/15 CRPS/37	a) T-cells b) FACS analysis c) IL-2, IFN-γ, IL-4, IL-10	No difference in percentage of cytokine producing cells between FMS and controls.	3d	2	0.6

Macedo, 2007 ACR	18/22	a) blood, PBMC b) automated biochip array; before and after 1.5 mg of dexamethasone per os c) IL-1α, IL-1β, IL-2, IL-4, IL-6, IL-8, IL-10, IFN-γ, TNF	After dexamethasone: reduction of cytokines FMS > controls.	3d	2	0.4

Ortega, 2010 ACR	9/9	a) PBMC b) ELISA c) IL-1b, TNF, IL-6, IL-10	For all cytokines investigated: higher values at baseline in FMS compared to controls; after aquatic exercise levels as in controls.	3d	3	0.3

Abbreviations:

ACR: American College of Rheumatology; CEMB: Center of Evidence Based Medicine; ELISA: enzyme linked immunosorbent assay; FMS: fibromyalgia syndrome; IL: interleukin; NOS: Newcastle Ottawa Scale; PBMC: peripheral blood mononuclear cells; R: receptor; RA: receptor antagonist; s: soluble; W-MeQS: Würzburg Methodological Quality Score; yr: year

PBMC of 151 FMS patients, 21 patients in disease control groups, and 159 healthy controls were investigated. All seven studies had a modified CEMB level of 3d [10,12,18,21,22,24,31]. One study reached a NOS score of 4 [12], two studies of 3 [24,31], and four studies of 2 [10,18,21,22]. Applying W-MeQS three studies were of medium methodological quality [10,21,31] and four were of low quality [12,18,22,24].

The following results were reproduced (regardless of the method used and with a minimum of two independent studies): ***no intergroup difference ***was found for IL-10 secretion from PBMC between FMS patients and controls [10,22,31].

FMS patients do not differ in PBMC cytokine levels from healthy controls.

#### Skin (Table 8)

Cytokine expression in skin of FMS patients was investigated in two studies including 116 FMS patients, and 59 healthy controls [15,27]. One study had a modified CEMB level of 3c [15] and one of 3d [27]; one study had a NOS score of 3 [16], the other of 2 [27]. Applying W-MeQS one study was of high methodological quality [16], one study of medium quality [27]. In the study by Salemi et al. [27] IL-1β, IL-6, and TNF were detectable in skin samples of a subgroup of FMS patients using reverse transcription PCR and immunohistochemistry, while in controls none of these cytokines could be detected. Blanco et al. [15] investigated TNF and MCP-1 in skin biopsies using immunohistochemistry and found lower MCP-1 expression in FMS patients than in controls. None of the results were reproduced.

**Table 8:** Characteristics of studies investigating levels of cytokines in skin of FMS patients.

**Author, yr**. Diagnosis criteria	N patients/controls	a) Material b) Methods c) Investigated targets	Results	Modified CEBM level	NOS	W-MeQS
Salemi, 2003 ACR	53/10	a) skin biopsy b) RT-PCR, IHC c) IL-1β, IL-6, TNF	Detectable cytokines in FMS: IL-1β (19/50) IL-6 (14/51) TNF (17/53) None of the cytokines could be detected in control skin.	3d	2	0.7

Blanco, 2010 ACR	63/49	a) skin b) IHC c) MCP-1, TNF	MCP-1: FMS < controls	3c	3	0.8

Abbreviations:

ACR: American College of Rheumatology; CEMB: Center of Evidence Based Medicine; FMS: fibromyalgia syndrome; IHC: immunohistochemistry; IL: interleukin; NOS: Newcastle Ottawa Scale; RT-PCR: reverse transcriptions PCR; W-MeQS: Würzburg Methodological Quality Score; yr: year

### Meta-analysis

In the selected 16 papers for meta-analysis authors investigated different cytokines with various methods. We therefore created subgroups of papers that investigated the same cytokine with the same or a comparable method. A group minimum of three studies was mandatory for meta-analysis.

A significant difference between controls and FMS was found only in two subgroups. When analyzing data of three studies on the pro-inflammatory cytokine IL-6 plasma levels investigated with ELISA or immunoassay or bioplex assay, patients with ***FMS had higher plasma levels of IL-6 ***compared to controls (SMD, -0.34; [-0.64, -0.03] 95% CI; p = 0.03; Table 4). There was also a trend for elevated serum IL-6 levels in FMS patients compared to controls (SMD, 1.01; [-0.03, 2.05] 95% [CI]; p = 0.06). No further intergroup differences were found even when regarding studies investigating serum, plasma, and whole blood as one group of "blood" (Table 9).

**Table 9:** Results of meta-analysis.

Study	FMS			Controls			Weight	SMD
	**Mean**	**Std**	**Total**	**Mean**	**Std**	**Total**		**IV, Random, 95% CI**

**IL-1: plasma, ELISA or immunoassay**								

Togo 2009	0.79	0.64	7	1.24	1.17	9	16.4%	-0.43 [-1.44, 0.57]

Feng 2009	74.5	116.5	100	45.6	53.1	35	41.3%	0.28 [-0.11, 0.66]

Bazzichi 2007	4.54	9.7	80	7.44	6.62	45	42.3%	-0.33 [-0.70, 0.04]

Total (95% CI)			187			89	100.0%	-0.10 [-0.58, 0.39]

Heterogeneity: Tau^2 ^= 0.11; Chi^2 ^= 5.52, df = 2 (P = 0.06); I^2 ^= 64%								

Test for overall effect: Z = 0.39 (P = 0.69)								

**IL-6: plasma, ELISA or immunoassay or bioplex assay**								

Togo 2009	14.0	10.9	7	16.8	15.3	9	9.6%	-0.19 [-1.19, 0.80]

Schwarz 2002	1.01	0.47	17	1.45	2.17	17	20.7%	-0.27 [-0.95, 0.40]

Bazzichi 2007	2.76	3.99	80	4.34	4.51	45	69.7%	-0.38 [-0.74, -0.01]

Total (95% CI)			104			71	100.0%	-0.34 [-0.64, -0.03]

Heterogeneity: Tau^2 ^= 0.00; Chi^2 ^= 0.15, df = 2 (P = 0.93); I^2 ^= 0%								

Test for overall effect: Z = 2.15 (P = 0.03)								

**IL-6: serum, ELISA or immunoassay or bioplex assay**								

Gür 2002	5.52	3.96	81	5.46	1.37	32	25.7%	0.02 [-0.39, 0.43]

Hernandez 2010	16.28	8.13	64	0.92	0.32	25	24.8%	2.20 [1.63, 2.77]

Wallace 2001	7.19	2.02	56	6.3	6.72	36	25.6%	0.20 [-0.22, 0.62]

Wang 2008	2.57	1.38	20	0.92	0.32	25	23.9%	1.71 [1.02, 2.40]

Total (95% CI)			221			118	100.0%	1.01 [-0.03, 2.05]

Heterogeneity: Tau^2 ^= 1.05; Chi^2 ^= 51.09, df = 3 (P < 0.00001); I^2 ^= 94%								

Test for overall effect: Z = 1.91 (P = 0.06)								

**IL-8: plasma, ELISA or immunoassay**								

Bazzichi 2007	61.89	149.0	80	7.9	17.5	45	41.2%	0.45 [0.08, 0.82]

Blanco 2010	83.0	253.0	79	84.0	253.0	59	42.8%	-0.00 [-0.34, 0.33]

Togo 2009	8.4	1.9	7	11.8	5.4	9	16.0%	-0.75 [-1.79, 0.28]

Total (95% CI)			166			113	100.0%	0.06 [-0.43, 0.56]

Heterogeneity: Tau^2 ^= 0.12; Chi^2 ^= 6.23, df = 2 (P = 0.04); I^2 ^= 68%								

Test for overall effect: Z = 0.25 (P = 0.80)								

**TNF: serum, ELISA or immunoassay or bioplex assay**								

Hernandez 2010	20.42	7.24	64	35.73	0.72	25	33.0%	-2.46 [-3.05, -1.87]

Wang 2008	3.27	5.08	20	3.89	12.95	80	33.5%	-0.05 [-0.54, 0.44]

Üçeyler 2006	7.41	11.01	26	6.7	9.5	40	33.5%	0.07 [-0.42, 0.56]

Total (95% CI)			110			145	100.0%	-0.81 [-2.31, 0.70]

Heterogeneity: Tau^2 ^= 1.70; Chi^2 ^= 49.74, df = 2 (P < 0.00001); I^2 ^= 96%								

Test for overall effect: Z = 1.05 (P = 0.29)								

**TNF: plasma, ELISA or immunoassay**								

Bazzichi 2007	22.59	29.55	80	11.07	6.77	45	28.0%	0.48 [0.11, 0.85]

Blanco 2010	163.0	435.0	79	161.0	319.0	59	28.5%	0.01 [-0.33, 0.34]

Feng 2009	12.2	10.2	100	40.1	73.5	35	27.6%	-0.73 [-1.12, -0.33]

Togo 2009	0.24	0.13	7	0.7	0.61	9	16.0%	-0.93 [-1.98, 0.13]

Total (95% CI)			266			148	100.0%	-0.21 [-0.82, 0.39]

Heterogeneity: Tau^2 ^= 0.30; Chi^2 ^= 21.71, df = 3 (P < 0.0001); I^2 ^= 86%								

Test for overall effect: Z = 0.69 (P = 0.49)								

Patients with FMS have higher plasma levels of IL-6 compared to controls. The discrepancy to the results of the systematic review (higher plasma IL-8 levels in patients with FMS) is due to the fact that only few studies were suitable for meta-analysis.

## Discussion

In this systematic review and meta-analysis we set out to clarify if cytokine profiles in FMS patients differ from controls analyzing published data available to date. Applying the modified CEBM levels, NOS, and W-MeQS we showed that the majority of the studies was of poor methodological quality. Our systematic review, in which we also considered study quality (modified CEBM level; NOS score; W-MeQS) shows that patients with FMS have higher serum IL-1RA, IL-6, and IL-8 levels compared to controls and that they also have higher plasma levels of IL-8. Looking at reproduced results (regardless of the method used and with a minimum of two independent studies) FMS patients do not differ from controls in serum IL-1β/IL-1, TNF, IL-2, IL-10, IFN-γ, and IL-4 levels; they also do not differ from controls in plasma IL-1β/IL-1, IL-6, TNF, IL-10, MCP-1, IP10, IL-2, and IFN-γ levels.

Our meta-analysis shows that patients with FMS have higher plasma levels of IL-6 compared to controls and a tendency for higher serum levels of IL-6 (p = 0.06). No further differences could be found.

IL-1RA, IL-6, and IL-8 are pro-inflammatory cytokines that may have algesic effects. The role of cytokines in the induction and maintenance of pain is well established in animal studies and also in pain syndromes [5]. Therefore the hypothesis that patients with FMS, who suffer from generalized pain may have an innate or acquired imbalance in cytokine production and secretion is plausible. Higher levels of these cytokines in plasma and/or serum of patients with FMS may be associated with pain in FMS. In line with these findings one study showed lower levels of the anti-inflammatory and analgesic cytokines IL-4 and IL-10 in FMS patients compared to healthy controls [30]. However, it remains elusive, whether these systemic changes in cytokine levels are the cause of pain in FMS or its consequence. Longitudinal studies are needed to answer this question.

The main problem in interpreting the results of studies on cytokines in FMS is that although cytokines have been investigated in a large number of studies, different methods have been used to analyze different cytokines in diverse material. Therefore a direct comparison of the results is hardly possible. Results obtained for one cytokine have mostly not been reproduced, the number of samples investigated per study was low (7/25 studies with n < 20 patients), and several studies report conflicting results - even in the same research group [13,14]. Also, possible confounding factors that physiologically may influence cytokine levels (e.g. circadian production; depression; physical activity; current infection) were not controlled in the majority of the studies.

Another problem is that the measurements mostly were not specifically hypothesis-driven, but were performed following the general assumption that "cytokines may play a role". Therefore in the majority of studies a large array of cytokines was screened without a clear hypothesis for each cytokine. This might be one reason why only few markers were repetitively analyzed, however, most markers were investigated only once - probably because they were part of a commercially available kit. In none of the studies a mechanistical approach was followed to investigate the mechanisms possibly underlying the effect of a specific cytokine in the induction and maintenance of defined symptoms of FMS. Therefore also the biological impact of the described changes in cytokine levels remains elusive. The question is also if in all studies the most appropriate method was used to detect the cytokine of interest. The majority of cytokines have a low expression and it is essential to apply methods that are sensitive enough. In the last years high- and ultra-sensitive bioassays have been developed that reliably allow the determination of cytokine proteins in body fluids. Since in the majority of cytokine studies analyzed here the sensitivity of the used method was not given it is not clear if the many negative results reported are "real" or due to low method sensitivity.

One further point is that there is no validated scoring system for the quality of laboratory methods in research papers. Such tools, however, are needed to assess study results and to compare the results of different studies, especially when looking at conflicting results. Standardization of the way laboratory research is performed by following quality standards and its presentation is warranted. The quality score W-MeQS that we present here may be one possibility.

In many cases the data provided in the papers were not applicable for meta-analysis and could not be calculated from the data presented in the publication. This fact and the diversity of methods and materials investigated made it almost impossible to form subgroups for meta-analysis.

Cytokines form a "fragile" system that can be influenced by many factors. The following issues might help improving the quality and the yield of future cytokine research in FMS patients: 1) Cytokine secretion follows circadian rhythmicity; the time points of material asservation should be kept constant. 2) Cytokine secretion has a large variability; measurements should be performed on two sets of samples and at least two different methods should be used (e.g. investigation of gene expression and protein levels). 3) Cytokine levels can be influenced by many environmental factors and by medication; patients should therefore be off medication at the time of material asservation or the possible influence of medication should be considered when interpreting the results. 4) Detailed patient characteristics (including disease duration; pattern of symptoms etc.) are essential for correlation of the cytokine measurements with clinical data. 5) The formulation of a clear literature-based hypothesis is warranted at the beginning of the cytokine study and a power calculation should be performed in advance. 6) Systemic and local cytokine expression should be investigated with appropriate methods. To choose the appropriate method, the expected level of expression should be taken into account and it should be made sure that the sensitivity of the method used is high enough to measure what is to be measured.

## Conclusions

The major consequence of our review is that cytokine research in FMS pathophysiology needs substantial improvement. More hypothesis-based and mechanistic studies are needed to understand if distinct cytokines are involved in causing symptoms of FMS or if they may be used as biomarkers of FMS symptoms. There is also a need for defined and applied quality standards for study design and performance in bench-side research and for standardization rules for study reports and manuscripts (like STROBE for clinical research). Possible confounding factors of cytokine levels should be controlled and a sufficient number of study subjects should be investigated e.g. following an a priori power calculation of sample size. Also the performance of prospective studies would be helpful.

## List of abbreviations

CEBM: Center for Evidence Based Medicine; FMS: fibromyalgia syndrome; GM-CSF: granulocyte macrophage colony-stimulating factor; IFN: interferon; IL: interleukin; IP-10: interferon gamma-induced protein-10; NOS: Newcastle-Ottawa Scale; MCP-1: monocyte chemoattractant protein-1; MIP-1: macrophage inflammatory protein-1; PBMC: peripheral blood mononuclear cells; R: receptor; RA: receptor antagonist; s: soluble; SMD: standardized mean differences; TGF: transforming growth factor; TNF: tumor necrosis factor-α; W-MeQS: Würzburg Methodological Quality Score.

## Competing interests

The authors declare that they have no competing interests.

## Authors' contributions

NÜ performed literature search, extracted and analyzed the data, generated Tables and Figures, and prepared manuscript. WH participated in data analysis and manuscript preparation. CS participated in literature search, data extraction, data analysis and manuscript preparation. All authors have read and approved the final manuscript before submission for peer review.

## Pre-publication history

The pre-publication history for this paper can be accessed here:

http://www.biomedcentral.com/1471-2474/12/245/prepub
